# Metal-rich organic matter and hot continental passive margin: drivers for Devonian copper-cobalt-germanium mineralization in dolomitized reef-bearing carbonate platform

**DOI:** 10.1007/s00126-022-01123-1

**Published:** 2022-06-01

**Authors:** Nicolas J. Saintilan, Corey Archer, Colin Maden, Elias Samankassou, Stefano M. Bernasconi, David Szumigala, Zach Mahaffey, Andy West, Jorge E. Spangenberg

**Affiliations:** 1 Institute of Geochemistry and Petrology, ETH Zürich, Clausiusstrasse 25, 8092 Zurich, Switzerland; 2 Department of Earth Sciences, Rue des Maraîchers 13, 1205 Geneva, Switzerland; 3 Geological Institute, ETH Zürich, Sonneggstrasse 5, 8092 Zurich, Switzerland; 4 Ambler Metals LLC, 3700 Centerpoint Drive, Ste. #101, Anchorage, AK USA; 5 Institute of Earth Surface Dynamics, University of Lausanne, Building Geopolis, 1015 Lausanne, Switzerland

**Keywords:** Rhenium-osmium, Bornite, Molybdenum, Algae, Bio-assimilated critical metals

## Abstract

The abundance and types of reef-bearing carbonate platforms reflect the evolution of Devonian climate, with conspicuous microbial-algal reefs in the warm Early and Late Devonian and sponge-coral reefs in the cooler Middle Devonian. A dolomitized Wenlock-Lower Devonian microbial-algal reef-bearing carbonate platform hosts epigenetic copper-cobalt-germanium (Cu-Co-Ge) sulfide mineralization at Ruby Creek-Bornite in the Brooks Range, Alaska. Here, we present rhenium-osmium (Re-Os) radiometric ages and molybdenum and sulfur (δ^98/95^Mo = +2.04 to +5.48‰ and δ^34^S = −28.5 to −1.8‰) isotope variations for individual Cu-Co-Fe sulfide phases along the paragenetic sequence carrollite-bornite-pyrite. In the context of a hot, extensional passive margin, greenhouse conditions in the Early Devonian favored restriction of platform-top seawater circulation and episodic reflux of oxidized brines during growth of the carbonaceous carbonate platform. Molybdenum and sulfur isotope data signal the stepwise reduction of hot brines carrying Cu during latent reflux and geothermal circulation for at least ca. 15 million years from the Early Devonian until Cu-Co sulfide mineralization ca. 379–378 million years ago (Ma) in the Frasnian, Late Devonian (weighted mean of Re-Os model ages of carrollite at 379 ± 15 Ma [*n* = 4]; Re-Os isochron age of bornite at 378 ± 15 Ma [*n* = 6]). On the basis of petrographic relationships between sulfides and solid bitumen, and the Mo and S isotope data for sulfides, we imply that the endowment in critical metals (e.g., Co, Ge, Re) in the Ruby Creek-Bornite deposit is linked to the activity of primary producers that removed trace metals from the warm Early Devonian seawater and concentrated Co, Ge, and Re in algal-bacterial organic matter in carbonate sediments.

**Supplementary Information:**

The online version contains supplementary material available at 10.1007/s00126-022-01123-1.

## Introduction

**Fig. 1:**
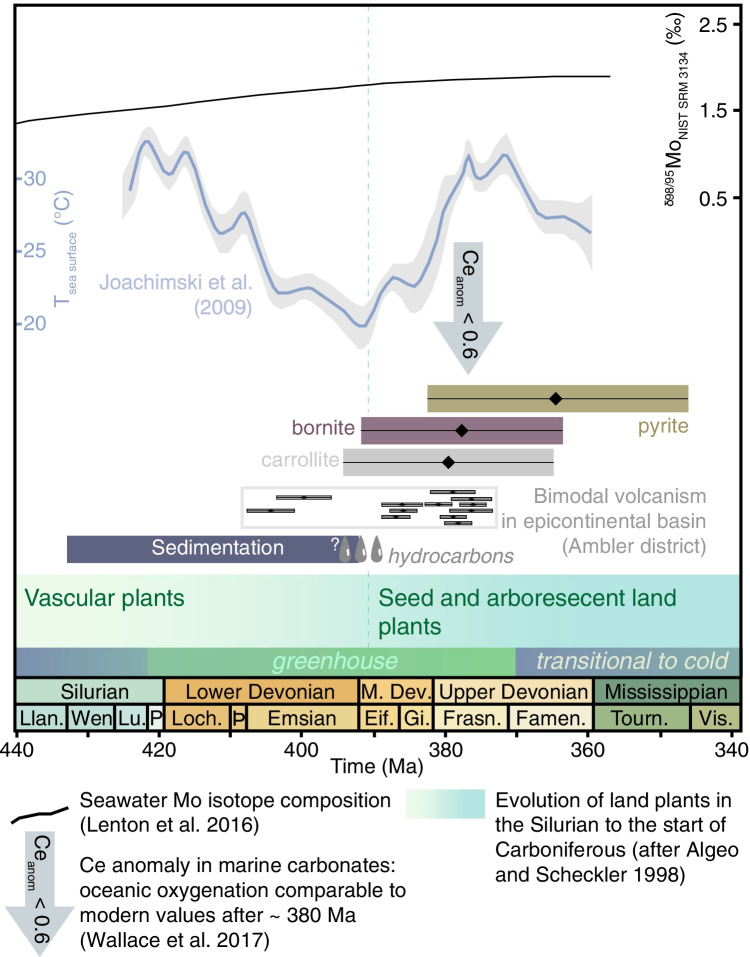
Chronology of stages of copper-cobalt (Cu-Co) mineralization at Ruby Creek-Bornite (new Re-Os isochron and model ages, this study; see ESM Fig. 1) in dolomitized carbonaceous limestone in a hot passive margin setting in the global context of ocean oxygenation and the rise of land plants in the Devonian (after Algeo and Scheckler 1998; Lenton et al. 2016; Wallace et al. 2017)

**Fig. 2:**
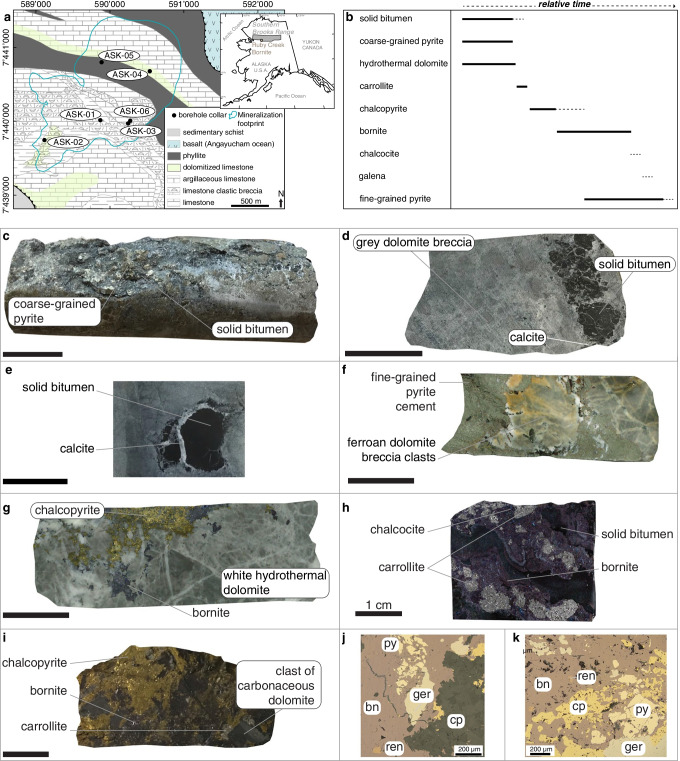
**a** Geological map of the Cosmos Hills in the area of the Ruby Creek-Bornite (RCB) Cu-Co deposit (source: Ambler Metals LLC and after Till et al. 2008) as well as its location in Northern Alaska (inset). The currently known footprint (~ 2,500 m × 1,500 m) of the deposit is projected to surface. The collars of the boreholes from which samples ASK-01 to ASK-06 were collected and analyzed in the present study are shown. See ESM Table 1 for a list and geographical information of the samples. **b** Paragenetic sequence at the Ruby Creek-Bornite deposit based on the studied samples. **c**–**e** Grey, clast-supported dolomite breccia is impregnated by solid bitumen as clots or films with haloes of white calcite. Solid, black to dark brown solid bitumen (insoluble in organic solvents) may be intergrown with coarse-grained subhedral to euhedral pyrite. **f** Fine-grained pyrite (sample ASK-02), which is in part coeval with bornite precipitation and may be distributed in the outer zones of RCB, cemented breccia of rounded and sub-angular clasts of ferroan dolomite associated with barium silicates. **g** Veins of and disseminated bornite and chalcopyrite, locally replaced angular clasts of light grey to white hydrothermal dolomite (ASK-05). The latter resulted from hydraulic brecciation of earlier grey dolomite, including veinlets in anastomosing networks. Brecciation and veining preceded and were coeval with chalcopyrite and bornite. **h**, **i** Coarse-grained grey carrollite (Cu[Co,Ni]_2_S_4_) cemented solid bitumen and clasts of dolomitized carbonaceous limestone before cementation by chalcopyrite and/or bornite with accessory chalcocite. Carrollite could have formed from the breakdown of pyrite (Hitzman 1986), associated with organic matter in carbonaceous limestone (ASK-06 and ASK-03). **j**, **k** Germanite (Cu_13_Fe_2_Ge_2_S_16_) after chalcopyrite, extensively replaced by bornite. Renierite (Cu_11_Ge_2_Fe_4_S_16_) as < 100-μm-large clots disseminated in bornite where the latter is intimately associated with solid bitumen and/or carbonaceous dolomite (reflected light microphotographs from Fig. 2i). Unless specified all scale bars are 2 cm long. Abbreviations: py: pyrite, bn: bornite, ger: germanite, ren: renierite, cp: chalcopyrite

The Devonian period was a warm greenhouse interval (Joachimski et al. 2009) that witnessed the largest reef constructions in Earth history (Copper and Scotese 2003) and the colonization of land plants (Algeo and Scheckler 1998; Lenton et al. 2016; Wallace et al. 2017; Fig. 1). The Devonian reef record reflects the evolution of Devonian climate (Joachimski et al. 2009). With warm to very warm sea surface temperatures in the Early and Late Devonian (Joachimski et al. 2009; Brugger et al. 2019), microbial-algal reefs were latitudinally widespread, i.e., 40°N to 46°S in the Lochkovian (416–411 Ma) and 45°N to 38°S in the late Frasnian-Famennian (377–359 Ma). Conversely, coral-stromatoporoid sponge reefs formed during the cooler Middle Devonian in water with intermediate tropical sea surface temperatures (Joachimski et al. 2009). Here, we focus on a dolomitized Wenlock-Lower Devonian microbial-algal reef-bearing carbonate platform in the Cosmos Hills, Southern Brooks Range, Alaska, USA (Fig. 2a and inset). This platform is host to the Ruby Creek-Bornite (RCB) copper-cobalt-germanium (Cu-Co-Ge) sulfide deposit associated with dolomite and post-oil solid bitumen (Hitzman 1986; Fig. 2b-k). In the context of reef expansion in the Devonian, we view the RCB deposit as a potential archive of the loops and feedbacks in the co-evolution of solid Earth, climate, and biosphere impacting on the flux of metals in the continental crust. Thus, this study is designed to explore the timing, origin, and source of metals in the RCB deposit in the greater context of the Devonian greenhouse world. To this end, we used (1) rhenium-osmium (Re-Os) isotope geochronology of individual sulfide species, and (2) transition metal abundances and molybdenum isotope composition and (3) sulfur isotope composition of those same sulfide species. Our interpretations focus on (1) the first-order controls on the flow, pulses, and redox evolution of mineralizing basinal brines, and (2) the specific metal endowment of the RCB deposit in critical metals (e.g., Co, Ge, Ni) as a consequence of the breakdown of metal-rich algal–bacterial organic matter during dolomitization and sulfide mineralization.

## Geological context and geodynamic setting in the Late Silurian to Late Devonian

**Fig. 3:**
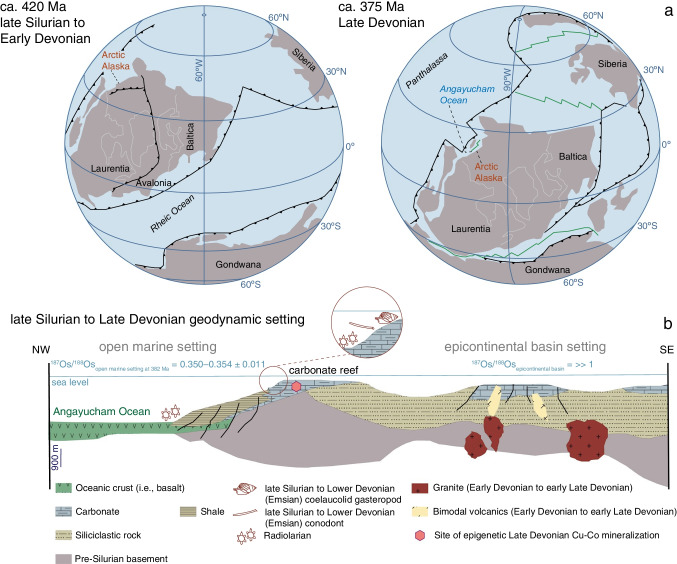
**a** Plate reconstruction and paleogeography of Alaska from the Late Silurian to the Late Devonian (after Robinson et al. 2019). **b** Paleogeodynamic setting of the Ambler district as an epicontinental basin at the time of opening of the Angayucham Ocean at ca. 375 Ma (after Hitzman et al. 1986)

The Scandian phase of the northern evolution of the Caledonian orogeny marked the final collision between Baltica and Laurentia (Miller et al. 2011; Gee et al. 2013; Robinson et al. 2019; Fig. 3a). At the end of convergence heralded by the Caledonian collision, a basin-and-range style of evolution commenced (Robinson et al. 2019). The Middle to Late Devonian rifting scenario involved crustal extension, thinning of the lithosphere, and incorporation of rift-related basin detritus in mantle-derived magmatism, as shown by zircon petrochronology (Miller et al. 2011; Hoiland et al. 2017; Robinson et al. 2019). Widespread extension triggered the opening of an epicontinental basin riddled by Middle to Late Devonian bimodal volcanism and associated granitic batholiths (Hitzman et al. 1986; Fig. 3a and 3b). This geodynamic configuration resulted in the opening of the Angayucham Ocean outboard of this northwest-facing epicontinental basin throughout the end of the Paleozoic and early Mesozoic (Hoiland et al. 2017; Robinson et al. 2019; Fig. 3a and 3b). A reef-bearing carbonate platform was built on a horst between the incipient Angayucham Ocean and this epicontinental basin on the seaboard of the extensional margin in Late Silurian (Wenlock—ca. 433 to 427 Ma) to Early Devonian (Emsian—ca. 407 to 393 Ma; Hitzman 1986; Hitzman et al. 1986; Till et al. 2008). Metazoan reefs rich in photozoans attest of shallow-marine water conditions in shelf zone with platform-top seawater circulation. Sustained warm air and sea surface temperatures (estimates of *T*_air surface_ ≥ 23 °C and *T*_sea surface_ = ca. 19–29 °C, at ca. 415 Ma and 40°N latitude; *T*_air surface_ ≥ 28 °C and *T*_sea surface_ = ca. 29 °C, at ca. 380 Ma and 20°N latitude; Joachimski et al. 2009; Brugger et al. 2019) favored carbonate reef building (Copper and Scotese 2003) in evaporitic sabkha setting on the edge of a shale-filled basin between 40°N latitude in the Late Silurian and 20°N latitude by the Late Devonian (Fig. 3a and 3b; Hitzman 1983, 1986; Hitzman et al. 1986; Till et al. 2008; Rohr et al. 2018; Robinson et al. 2019).

## The Ruby Creek-Bornite deposit

### Geology and mineral characteristics of a carbonate-hosted Cu-Co-Ge ore deposit

The RCB Cu-Co-Ge sulfide deposit is located in the Ambler Mining District on the southern flank of the Brooks Range of Alaska, USA (Fig. 2a and inset). The RCB deposit is a large carbonate-hosted Cu-Co-Ge sulfide deposit with indicated copper resources of 41.7 million tons (Mt) at an average grade of 1.04% Cu, with an additional inferred resource of 144.1 Mt at an average grade of 1.68% Cu. The inferred cobalt resource amounts to 185.8 Mt at an average grade of 0.02% Co (Sim et al. 2022). In comparison, at the Kipushi Cu-Co-Ge deposit in the Central African Copperbelt, to which the RCB deposit was compared (Hitzman 1986; see below), the mined orebodies contained 60 Mt grading 11.0% Zn, 6.8% Cu, and 0.3% Ge (Intiomale and Oosterbosch 1974; Tshileo et al. 2003; Heijlen et al. 2008).

The RCB deposit is hosted by dolomitized Wenlock-Lower Devonian carbonate rocks incorporated in the Brooks Range fold-and-thrust-belt resulting from Jurassic to Cretaceous deformation (Hoiland et al. 2017, 2018). Unlike other parts of the Cosmos Hills subjected to greenschist-facies metamorphism at 114 ± 5 Ma (U–Pb zircon laser ablation inductively coupled mass spectrometry; Hoiland et al. 2018), the rocks hosting the RCB mineralization underwent limited metamorphic overprint, brittle disruption and preserve the record of hydrothermal events (Hitzman 1986). The conodont alteration index of 5–6 suggests that temperature in the carbonate rocks remained below 360 °C (Till et al. 2008) throughout their geodynamic evolution. The rocks hosting the RCB deposit show an inward zonation from the outer carbonaceous limestone to a halo of low iron dolomite with disseminated and intergranular solid bitumen (Fig. 2c-e, “Dolomites A & B”, see below), around a core of ferroan dolomite (Fig. 2f) with intergranular solid bitumen. A fault-controlled and retreating hydrothermal front drove patchy dolomitization promoted by oxidation of organic matter and shaped by internal argillaceous limestone aquitards (i.e., “seals”) during early and burial diagenesis (Hitzman 1986). This pattern is compatible with multiple pulses of Fe–Mg-rich acidic hydrothermal fluids involved in (1) fluid-rock interaction and alteration of organic compounds (mainly hydrocarbons) increasing the dissolved inorganic carbon content, (2) dissolution and recrystallization of earlier dolomite phases up to 300 °C, and then (3) hydraulic brecciation in dolostone of low ductility (Hitzman 1986; Davies and Smith 2006; Fig. 2g).

Disseminated, fine-grained pyrite started mineralizing early in the paragenetic sequence (Bernstein and Cox 1986). An initial and pervasive dolomite that includes disseminated solid bitumen in intergranular voids and stylolites (“Dolomite A” of Hitzman 1986) overlies the most abundant dolomite facies in the mineralization (“Dolomite B” of Hitzman 1986). The latter occurs as irregular veins and breccias including clasts of dolomite A. Fine-grained pyrite is ubiquitous in dolomite B and most abundant at the gradational contact with dolomite A. Solid bitumen is coarse in dolomite B and fills intergranular voids (Hitzman 1986). The origin and crucial role of this organic matter in the mineralizing processes are discussed in the present work on the basis of the new petrographic and isotope data combined with previous interpretations by Runnells (1965) and Hitzman (1986).

Above the core of ferroan dolomite, massive bornite ± chalcocite-calcite mineralization is commonly located at the lower contacts of the shale aquitards in the low-iron dolomite B. Bornite-mineralized zones replaced massive coarse-grained, cobaltiferous pyrite with minor sphalerite that had formed below, and around, the shale aquitards. Those bornite-rich zones are surrounded by haloes of chalcopyrite-bornite-calcite ± dolomite ± carrollite (Cu[Co,Ni]_2_S_4_). Hydraulic brecciation of dolomite resulted in additional copper mineralization in the form of a dense network of bornite ± chalcopyrite-white dolomite-mineralized veins and stringers that cut across the ferroan and low iron dolomites (Fig. 2g), and even locally into the non-dolomitized carbonaceous limestone (Hitzman 1986).

Tennantite-tetrahedrite are closely associated with chalcopyrite and rarely found in bornite-rich zones. Anhedral grains of tennantite-tetrahedrite are intergrown with chalcopyrite in millimetre-thick veinlets (Bernstein and Cox 1986). Contrary to tennantite-tetrahedrite, zinc-bearing renierite (Cu_10_ZnGe_2_Fe_4_S_16_) and germanite (Cu_13_Fe_2_Ge_2_S_16_) are found almost exclusively in association with, and as inclusions in, bornite (Fig. 2j, k). Arsenian renierite (Cu_11_GeAsFe_4_S_16_) and vanadium-bearing germanite (with up to 3 wt% V) are specific to the RCB deposit (Bernstein and Cox 1986).

Cymrite, a micaceous barium silicate (BaAl_2_Si_2_(O,OH)_8_*H_2_O), is found as sheet-like crystals (1–2 vol%) with pseudomorphs of copper sulfides (Runnells 1964, 1969). Cymrite is most abundant in the core of iron-rich dolomite which is bereft of significant copper mineralization. The presence of Ba-silicate and the absence of abundant barite in the deposit with only rare deposition of barite late in the paragenetic sequence after copper sulfides (Runnells 1969) signals late availability of sulfate in the system.

### Is the RCB deposit unique?

The RCB deposit shares striking similarities with the Kipushi Cu-Co-Ge sulfide deposit in the Central African Copperbelt (Heijlen et al. 2008) and the Otavi Mountain Land district (including the Tsumeb Cu deposit) in Namibia (Chetty and Frimmel 2000). These large deposits have in common (1) features of diagenetic to epigenetic sulfide mineralization, (2) emplacement in carbonate stratigraphy, and (3) early pyrite-dolomite alteration of the host limestone followed by copper dominant sulfide mineralization. All these deposits occur in intra-continental to continental margin settings characterized by extensional tectonics and bimodal volcanism (Chetty and Frimmel 2000; Hitzman et al. 2010). Basin-margin faults seem to play an important role in localizing mineralizing fluids (Hitzman et al. 2010). Yet, the canonical interpretation of lithological break as syn-sedimentary faults was recently challenged (Turner et al. 2018; Spinks et al. 2021). Lithological breaks associated with sulfide mineralization were reinterpreted at Kipushi as steep depositional margin of carbonate lithofacies where carbonate build-up focused mineralizing fluids when in contact with overlying fine-grained terrigenous strata (e.g., shale and siltstone aquitards; Turner et al. 2018). The critical control of carbonate lithofacies is highlighted further at the RCB and Kipushi deposits where the Cu-Co-Ge sulfide orebodies are intimately associated with solid bitumen in dolomite (Runnells 1964, 1965, 1969; Hitzman 1986; Heijlen et al. 2008). Given this detailed knowledge for carbonate-hosted Cu-Co-Ge deposits, our present study explores the controls exerted on the mineralizing processes at Ruby Creek-Bornite by (1) a hot continental passive margin setting and (2) the chemical and redox potential of carbonaceous limestone in a reef-bearing platform on this continental margin. Our goal is to contribute advances that can be utilized to build a greater understanding of the origin of Ruby Creek-Bornite-Kipushi-type ore deposits.

## Results

### Paragenetic relationships of sulfides, solid bitumen, and dolomite

Detailed ore textures and mineralogy are provided in Runnells (1969) and Hitzman (1986). The petrographic observations and paragenetic relationships (Fig. 2b-k) presented here pertain to the samples that were used to contextualize the new sulfide-specific radiogenic and stable isotope data. Grey, clast-supported dolomite breccia is impregnated by solid bitumen as coalesced clots and globules, or films with haloes of white calcite (Fig. 2c-e). Solid, black to dark brown solid bitumen (insoluble in organic solvents) may be intergrown with coarse-grained subhedral to euhedral pyrite (Fig. 2c). Fine-grained pyrite (sample ASK-02), which is in part coeval with bornite precipitation and may be distributed in the outer zones of RCB, cements breccia of rounded and sub-angular clasts of ferroan dolomite associated with barium silicates (Fig. 2f). Veins and disseminated bornite and chalcopyrite cement and locally replace angular clasts of light grey to white hydrothermal dolomite (sample ASK-05). The latter resulted from hydraulic brecciation of earlier grey dolomite, including veinlets in anastomosing networks. Brecciation and veining preceded and were coeval with chalcopyrite and bornite (Fig. 2g). Coarse-grained grey carrollite cemented solid bitumen and clasts of dolomitized carbonaceous limestone before cementation by chalcopyrite and/or bornite with accessory chalcocite (samples ASK-06 and ASK-03; Fig. 2h and i). In these sulfide-rich samples associated with solid bitumen and clasts of carbonaceous limestone, germanite, which formed coevally with and after chalcopyrite, was included and locally replaced by bornite (Fig. 2j and k). In those same samples, renierite is found as < 100-μm-large clots that are disseminated in bornite.

#### Radiogenic and stable isotope geochemistry of Late Devonian sulfides

We complement a preliminary Re-Os date (384 ± 4 Ma) produced by using data points of multiple sulfide phases (bornite, chalcopyrite, pyrite from ore dump samples) in a single isochron regression in the ^187^Re versus ^187^Os space (i.e., by considering an initial [^187^Os/^188^Os]_i_ ratio of 0.3 ± 5.5; Selby et al. 2009). Here, we use the new method of Re-Os isotope geochemistry and isochron regression applied to mineral separates of individual sulfide species co-existing in single hand samples (carrollite, bornite, pyrite in this paragenetic order; Fig. 2b, Electronic Supplementary Materials—ESM Table 1) but analyzed separately (Saintilan et al. 2018, 2020). The δ^34^S signatures of these mineral separates were constrained by standard techniques whereas variations in the δ^98/95^Mo of purified Mo fractions of each sulfide species were determined by multicollector inductively coupled mass spectrometry. Extended methods, explanation of data handling, and diagrams of Re-Os isochron and model ages are provided in the ESM.

Rhenium and total Os (Os_total_) concentrations in the analyzed sulfides are high (59.2 to 642.4 ng g^−1^ and 274 to 2,933 pg g^−1^, respectively; Table 1). Common Os contents, estimated using abundances in ^192^Os, represent a negligible fraction of Os_total_ (11 to 113 pg g^−1 192^Os). The elevated ^187^Re/^188^Os values (9,615–13,521) that are positively correlated with highly radiogenic ^187^Os/^188^Os ratios (59.3–87.5) are diagnostic of a total ^187^Os budget that largely comprises radiogenic ^187^Os^*^ (95.0–98.8%) with minor correction for common ^187^Os_c_. Carrollite (527.5–642.4 ng g^−1^ Re; 2,429–2,933 pg g^−1^ Os_total_; 36.9–61.1 μg g^−1^ Mo; Tables 1 and 2) and bornite (393.7–472.3 ng g^−1^ Re; 1,735–2,135 pg g^−1^ Os_total_; 21.3–28.9 μg g^−1^ Mo) cementing solid bitumen and fragments of carbonaceous limestone (Fig. 2h, i) have the highest Re, Os_total_, and Mo contents. The sulfides span a large range of isotopically heavy Mo isotope compositions with carrollite (+ 3.78 to + 3.89‰) and bornite (+ 2.21 to + 5.49‰) being isotopically heavier than pyrite (+ 2.04 to + 2.24‰; Table 2 and ESM Table 2).

**Table 1:** Synopsis of the Re-Os isotope geochemistry data for bornite, carrollite, and pyrite at Ruby Creek-Bornite, Southern Brooks Range, AK

Name	Mineral	Aliquot size	Re	± 2 s	^187^Re	± 2 s	Total Os	± 2 s	^192^Os	± 2 s	^187^Os^*^	± 2 s	%^187^Os^*^	^187^Re/^188^Os	± 2 s	^187^Os/^188^Os	± 2 s	*rho*	% Re_blk_	%^187^Os_blk_	%^188^Os_blk_	Os_i_ at isochron age^a^	± 2 s	Model age^b^	± 2 s
		(mg)	(ng/g)		(ng/g)		(pg/g)		(pg/g)		(pg/g)		(%)											(Ma)	
004–01-ASK-01	Bornite	53.10	142.89	0.20	89.81	0.13	649	7	26.6	0.1	564	41	96.3	10,694	36	69.7	0.4	0.520	0.021	0.016	0.858	2.35	0.45	377	32
007–01-ASK-01	Bornite	55.74	146.16	0.16	91.86	0.10	662	7	26.1	0.1	578	40	96.5	11,139	41	72.7	0.4	0.614	0.015	0.007	1.176	2.53	0.48	378	31
008–01-ASK-01	Bornite	54.89	142.13	0.19	89.34	0.12	643	6	25.7	0.1	561	39	96.4	10,985	32	71.5	0.3	0.559	0.042	0.007	1.211	2.31	0.37	377	31
004–03-ASK-03	Bornite	80.89	452.01	0.52	284.11	0.32	2,042	17	76.7	0.1	1,797	117	96.6	11,721	25	76.7	0.3	0.429	0.004	0.003	0.197	2.87	0.33	379	29
006–03-ASK-03	Bornite	59.00	472.27	0.64	296.84	0.40	2,135	20	80.2	0.2	1,878	123	96.6	11,709	35	76.7	0.3	0.560	0.006	0.007	0.179	2.89	0.41	379	29
008–04-ASK-04	Bornite	62.13	98.12	0.11	61.67	0.07	433	5	14.4	0.1	387	22	97.1	13,521	54	87.5	0.5	0.694	0.054	0.010	1.894	2.27	0.59	376	25
005–08-ASK-06	Bornite	59.55	403.31	0.59	253.49	0.37	1774	20	73.9	0.3	1,538	113	96.2	10,856	44	68.4	0.4	0.591	0.007	0.005	0.277	0.03	0.50	364	32
007–06-ASK-06	Bornite	57.97	393.71	0.41	247.46	0.26	1735	15	72.6	0.2	1,504	111	96.2	10,793	27	68.1	0.3	0.599	0.005	0.003	0.410	0.41	0.15	364	32
005–06-ASK-05	Bornite	75.59	59.21	0.16	37.22	0.10	274	5	10.9	0.2	239	18	96.4	10,784	180	71.8	2.1	0.552	0.036	0.026	1.458	3.86	2.40	385	33
006–02-ASK-02	Pyrite	70.26	248.04	0.30	155.90	0.19	1,103	6	47.0	0.1	947	81	95.6	10,505	17	66.8	0.1	0.410	0.009	0.011	0.256	n.a	n.a	367	32
007–02-ASK-02	Pyrite	75.14	324.16	0.26	203.75	0.17	1,414	13	52.1	0.2	1,241	90	96.2	12,385	36	78.4	0.4	0.619	0.005	0.002	0.441	n.a	n.a	367	28
008–02-ASK-02	Pyrite	56.85	220.56	0.23	138.63	0.14	964	7	45.6	0.1	813	79	95.0	9,615	21	59.3	0.2	0.558	0.026	0.005	0.663	n.a	n.a	354	36
006–05-ASK-06	Carrollite	57.55	642.40	0.87	403.77	0.55	2,933	28	113.3	0.3	2,632	234	98.8	11,276	35	74.4	0.4	0.569	0.004	0.005	0.130	n.a	n.a	382	30
006–06-ASK-06	Carrollite	62.31	527.54	0.68	331.57	0.42	2,429	17	97.8	0.2	2,169	202	98.8	10,728	23	71.1	0.2	0.527	0.005	0.006	0.139	n.a	n.a	383	32
008–06-ASK-06	Carrollite	62.21	548.15	0.55	344.53	0.35	2,478	17	101.4	0.2	2,209	209	98.8	10,758	19	69.8	0.2	0.531	0.010	0.002	0.274	n.a	n.a	375	32
008–07-ASK-06	Carrollite	59.17	569.85	0.60	358.17	0.38	2,575	18	103.2	0.2	2,301	213	98.8	10,983	20	71.4	0.2	0.483	0.010	0.002	0.283	n.a	n.a	376	31

^a^Calculated with a model age of 378 ± 15 Ma for bornite aliquots

^b^Model age calculated with correction for common Os using ^187^Os/^188^Os_(initial)_ of 2.40 ± 2.86 from the Model 3 isochron for bornite

*n.a*. not applicable

**Table 2:** Summary of the transition metal abundances (nickel and molybdenum) and molybdenum and sulfur isotope data for bornite, carrollite, and pyrite at Ruby Creek-Bornite, Southern Brooks Range, AK

Sample	Mineral	Petrographic texture	Sample weight	Ni	Mo	δ^34^S V-CDT	δ^98/95^Mo V-NIST_SRM3134_ (Batch #1)
			(g)	(μg g^−1^)	(μg g^−1^)	(‰ ± 2 standard error)	(‰)
ASK-02a	Pyrite	Cement of ferroan dolomite	0.03714	138	8.1	− 2.7 ± 0.3 (*n* = 2)	+ 2.22
ASK-02b	Pyrite		0.07538	190	10.1		+ 2.13
ASK-06_CAR_a	Carrollite	Cementing solid bitumen and fragments of dolomitized carbonaceous limestone	0.07432	34,077	61.1	− 1.8 ± 0.4 (*n* = 6)	+ 3.78
ASK-06_CAR_b	Carrollite		0.05320	69,063	36.9		+ 3.86
ASK-01	Bornite	Replacing coarse-grained pyrite and associated with solid bitumen	0.02899	1.3	13.9	− 28.5 ± 0.4 (*n* = 3)	+ 2.44
ASK-03	Bornite	Cementing solid bitumen and fragments of dolomitized carbonaceous limestone	0.02114	13.5	28.9	− 3.3 ± 0.2 (*n* = 3)	+ 5.48
ASK-04	Bornite	Associated with white hydrothermal dolomite and cementing clasts of dolomite matrix	0.02856	1.3	15.6	− 2.8 ± 0.5 (*n* = 6)	+ 2.75
ASK-05	Bornite	Associated with white hydrothermal dolomite	0.06387	0.14	3.3	− 7.8 ± 0.3 (*n* = 2)	+ 2.21
ASK-06_Bn	Bornite	Cementing solid bitumen and fragments of carbonaceous limestone	0.03934	123	21.3	− 3.6 ± 0.3 (*n* = 2)	+ 3.53

Preserved Re-Os systematics of six bornite aliquots (δ^34^S =  − 28.5 ± 0.4‰ to − 2.8 ± 0.5‰; Table 2) yield a Model 3 Re-Os isochron date of 378 ± 15 Ma that accounts for potential variability in the initial ^187^Os/^188^Os (Os_i_) of each aliquot within the 2.40 ± 2.86 range determined by isochron regression (ESM Fig. 1). These bornite aliquots have individual model ages at ca. 379–376 Ma whereas three additional aliquots of bornite that are positioned slightly above (δ^34^S =  − 7.8 ± 0.3‰) and below (δ^34^S =  − 3.6 ± 0.3‰) the 378-Ma isochron have slightly older (ca. 385 Ma) and younger (ca. 364 Ma) model ages, respectively. The Re-Os data of single carrollite aliquots (*n* = 4; δ^34^S =  − 1.8 ± 0.4‰) return model ages between 383 and 375 Ma and a weighted mean average at 379 ± 15 Ma. Similarly, individual aliquots of fine-grained pyrite cement (*n* = 3; δ^34^S =  − 2.7 ± 0.3‰) have Re-Os data yielding model ages between 367 and 354 Ma and a weighted mean average at 364 ± 18 Ma (ESM Fig. 1).

## Discussion and conclusions

### Large-scale fluid flow and tectonic controls on Late Devonian mineralization

**Fig. 4:**
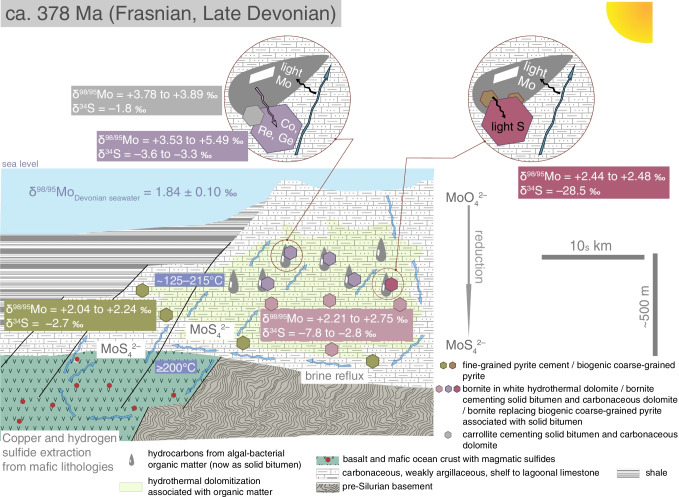
Genetic model for epigenetic Cu-Co mineralization hosted in dolomitized Wenlock-Emsian carbonaceous limestone at Ruby Creek-Bornite in the Frasnian to Famennian

In the extensional tectonic setting of a carbonate shelf at the margin of an epicontinental basin (Fig. 3b), basin dewatering contributed active reflux of fluids derived from evaporitic brines in the host reefal, Wenlock to Emsian carbonate platform (Hitzman 1986). With high heat flow sustained by bimodal volcanic activity in the adjacent epicontinental basin from ~ 405 to 376 Ma (U–Pb zircon geochronology; McClelland et al. 2006; Raterman et al. 2006; Figs. 1 and 3b), brine-derived fluids then circulated via a combination of latent reflux (Jones et al. 2002) and geothermal circulation for at least 15–30 million years from the late Emsian to ca. 379–364 Ma (Re-Os time stamps for Cu-Co sulfide mineralization, including pyrite; Fig. 1). The brine fluids with salinities at 7 to 13 wt% eq. NaCl (2 to 4 times average Cambrian–Devonian seawater salinity; Demicco et al. 2005) could produce matrix dolomite at temperatures as high as 300 °C and then cooled to precipitate Cu sulfides at ~ 120–225 °C (Hitzman 1986). Molybdenum isotope variations in sulfides with high Mo contents (3.3 to 61.1 μg g^−1^ Mo) trace the hydrological cycle of brine fluids encountering an increasingly anoxic environment (Ryb et al. 2009), until formation of reduced sulfide-bearing and copper-rich fluids (Fig. 4). The conditions, which led to the involvement of reduced sulfide-bearing and copper-rich fluids in the mineralizing processes, are explained below. In brines derived from Mo-rich (> 50 µg g^−1^) Devonian seawater (Scott et al. 2008; δ^98/95^Mo_seawater_ =  + 1.84 ± 0.10‰; Gordon et al. 2009), Mo remains in solution as molybdate [MoO_4_^2–^] under oxic conditions (Ryb et al. 2009). The progressive and incomplete sulfidation of [MoO_4_^2–^] to tetrathiomolybdate [MoS_4_^2–^] in brine is accompanied by net residual fluid positive isotope fractionation during subsurface brine migration (Ryb et al. 2009; Kerl et al. 2017). This process is recorded in the Mo isotope composition of the pyrite cement (8–10 μg g^−1^ Mo; δ^98/95^Mo =  + 2.04 to + 2.24‰; Fig. 4) of ferroan dolomite that precipitated at ca. 364 Ma once the geothermal gradient was insufficient for brines to be hot enough to transport copper in solution. These temporal and geochemical constraints imply that the hydrological cycle evolved in space and time with the earlier inflow of reduced sulfide-bearing and copper-rich brines at ca. 379–378 Ma (Fig. 4) that caused hydraulic fracturing and cementation of angular to sub-angular fragments of matrix dolomite by white hydrothermal dolomite and Cu-Co sulfides (Fig. 2g). By consuming isotopically heavy MoS_4_^2–^ complexes in subsurface reducing conditions, bornite precipitated with heavy Mo isotope signatures (δ^98/95^Mo =  + 2.21 to + 2.75‰; Fig. 4). The latter and the dominant sulfur isotope compositions of Cu-Co sulfides and pyrite in the RCB (δ^34^S =  − 3.6 to − 1.8‰) are compatible with leaching of copper, sulfur, and additional isotopically heavy Mo from magmatic sulfides (Voegelin et al. 2012) in newly formed oceanic crust flooring the shale-filled basin adjacent to the carbonate platform. In this context, we suggest that high heat gradient conditioned the co-transport of copper and reduced sulfur species in solution at moderate temperatures (≥ 200–225 °C; Zhong et al. 2015). Furthermore, Cu-Co sulfides are present at the contact of solid residues of post-oil solid bitumen and coarse-grained pyrite in the dolomitized carbonaceous limestone (Fig. 2h, i). The interaction of relatively reduced, copper-rich brines with organic matter and additional precipitation of Cu-Co sulfides using isotopically light, biogenic hydrogen sulfide (Runnells 1969; δ^34^S =  − 28.5 to − 7.8‰, this study) explains an additional net residual fluid positive fractionation of Mo isotopes: the light Mo isotopes were scavenged by organic compounds (Ryb et al. 2009; King et al. 2018) resulting in the heaviest Mo isotope compositions in Cu-Co sulfides (δ^98/95^Mo_bornite_ =  + 2.44 to + 5.49‰; δ^98/95^Mo_carrollite_ =  + 3.78 to + 3.89‰; Fig. 4).

Our findings highlight the importance of ore-forming processes associated with brine-derived fluid flow in organic-rich carbonate lithofacies during the evolution of a Late Silurian to Devonian hot continental margin. In fact, a remarkable feature of the Devonian worldwide may be that of ore-forming processes involving either (1) large-scale flow of heated evaporitic basinal brines, and/or (2) strong controls on sub-surface sulfidic conditions by the extent of organic carbon burial in continental margin (e.g., Mako and Shanks III 1984; Wilkinson et al. 2005; Magnall et al. 2018; Gadd et al. 2020; Saintilan et al. 2022). For the Late Devonian specifically, it is suggested that high primary productivity and enhanced organic carbon burial were key processes for sulfidic trap sites for metals in sub-surface conditions (Magnall et al. 2018). In the next section, we develop a model in which primary producers (Falkowski and Knoll 2011) in Early Devonian seawater acted as efficient metal traps. In this model, we further explain how organic carbon burial in platformal carbonaceous limestone not only optimized sub-surface sulfidic conditions, but also contributed to the metal endowment of the RCB deposit.

### Supplementary biogenic metal source

Textural evidence suggests that solid bitumen is derived from tiny particles of organic matter that were endogenous to the carbonate sediments that lithified into the carbonaceous limestone hosting the RCB deposit (Runnells 1965). The positive spatial relationship between solid bitumen and recrystallized, clear dolomite (Fig. 2d, e) suggests that recrystallized dolomite results from the removal of tiny particles of black carbonaceous matter that coalesced into liquid hydrocarbon endogenous to the host limestone. This process left recrystallized dolomite clear and commonly free of dust-size, finely disseminated carbonaceous matter (Runnells 1965; Hitzman 1986). The rarer type of solid bitumen, shaped like droplets, and filling vugs with a halo of white calcite (Fig. 2e; Runnells 1965; Hitzman 1986, this study) may represent areas where endogenous hydrocarbon flowed in liquid form into vugs within the host dolomite (Runnells 1965). In summary, all textural and geochemical evidence (Runnells 1965; Hitzman 1986, this study) favor a preferred interpretation that hydrocarbons were related to an endogenous source rather than hydrocarbons derived from a regional shale source rock. Runnells (1965) suggested that endogenous hydrocarbon moved in suspension in the basinal brines throughout dolomitization and sulfide mineralization.

In our model, we suggest that algal–bacterial organic matter endogenous to limestone not only contributed sub-surface sulfidic conditions favorable for sulfide mineralization but also acted as a specific source of critical metals in the RCB deposit. Indeed, in light of petrographic evidence and our new δ^98/95^Mo and δ^34^S data, we propose that the high Re contents, the variable Os_i_ of Cu-Co sulfides, and the association of Ge-sulfides consistently with Cu-Co sulfides (Fig. 2) relate to algal–bacterial primary producers in the warm Early Devonian seawater. These micro-organisms fed on Re, Co, Ge, and minor Os as catalyzers in their metabolic pathways until incorporation of this metal-rich organic matter in carbonate sediments and lithification to carbonaceous limestone. In details, Ge can be bio-assimilated by modern microalgae in surface waters (Sutton et al. 2010). Modern microalgae and bacteria utilize Co and Ni as alternatives to elements essential to their metabolism (Andersson et al. 2020; Hawco et al. 2020; Morel et al. 2020). Modern macroalgae accumulate Re preferentially over Os from seawater. The abundance of Re (10s of ppb) and Os (100s of ppt) in macroalgae is primarily controlled by uptake from the dissolved load in local seawater (Racionero-Gómez et al. 2016, 2017). The Wenlock–Lower Devonian reef-bearing carbonate platform developed at the interface between open marine conditions (^187^Os/^188^Os_seawater at 382 Ma_ ~ 0.350–0.354 ± 0.011; Fig. 3b; Saintilan et al. 2021) and an epicontinental basin under the influence of coastal inputs (Os_basin-rivers_ >  > 1; Peucker-Ehrenbrink and Ravizza 2000) combining the effects of erosion of the Caledonian orogen (Robinson et al. 2019) and the impact of land plants on chemical weathering of crustal sulfides and organic matter (Algeo and Scheckler 1998). Macroalgae do not fractionate Os isotopes and record variations in ^187^Os/^188^Os of seawater at the month to year scale (Racionero-Gómez et al. 2017). Therefore, we suggest that the high variability of the bornite Os_i_ (2.40 ± 2.86) is driven by original assimilation of Os by algae in the carbonate platform setting in the Devonian (Fig. 3b). In conclusion, we posit that the endowment in Co, Ge, and Re in the RCB deposit results from elemental exchange at the micro-scale between the hot mineralizing fluids and algal–bacterial organic matter in carbonaceous limestone of high chemical potential.

### Climax of Devonian greenhouse conditions in the Frasnian: a contribution to the flow of mineralizing fluids?

The age of the epigenetic Cu-Co sulfide mineralization at ca 379–378 Ma coincides with the ca. 380–375 Ma upslope to the climax of greenhouse climatic conditions in the Frasnian-Famennian (Fig. 1). We question whether this temporal overlap is a causal or a fortuitous relationship. In the context of a hot, extensional passive margin (Hitzman 1986; Hitzman et al. 1986), we discuss whether short-term transgression-regression pulses (Shalev and Yechieli 2007) in a warming Frasnian climate (Joachimski and Buggisch 2003; Joachimski et al. 2009) could have been an additional contribution to the up-and-down flux (Shalev and Yechieli 2007) of mineralizing brines in dolomitized carbonaceous limestone. Trangression-regression cycles induce “fluid pumping” that causes fluid pressure changes and modifies the flow of oxidized, evaporitic residual brines in marginal and rift basin (Vasyukova and Williams-Jones 2022), i.e., recession of sea level may cause brine discharge whereas rise of sea level makes brines sink through faults and migrate through tilted blocks (Shalev and Yechieli 2007).

The building of the reef-bearing carbonate platform that hosts the RCB deposit took place during a decline of sea level from the Late Silurian (Ludlow) through Early Devonian (Emsian) following a eustatic high in mid-Wenlock (Haq and Schutter 2008). The Middle Devonian coincided with the beginning of another long-term and continuous rise of sea level (Johnson et al. 1985), which reached its acme in the early Late Devonian (ca. 382 Ma; Frasnian; Haq and Schutter 2008). The timing of Cu-Co sulfide mineralization at ca. 379–378 Ma overlaps with the end of rapidly succeeding transgression-regression cycles that commenced at ca. 383 Ma (i.e., “depophase IIb” of Johnson et al. 1985) in this overall global transgression (Johnson et al. 1985; Kabanov and Jiang 2020). Under greenhouse conditions in the Late Devonian (Fig. 1), glacio-eustasy is non-existent and third-order sea level changes of 15–35 m in the Middle to Late Devonian are documented (Witzke 2011; Smith et al. 2019a, b; Kabanov and Jiang 2020) although sea-level changes up to 90–145 m from the late Middle Devonian (Givetian) to Late Devonian (Famennian) may have been possible (Witzke 2011; Ver Straeten et al. 2019). Therefore, in this context and considering the “fluid pumping” model (Shalev and Yechieli 2007; Vasyukova and Williams-Jones 2022), we conceptualize that succeeding transgression-regression cycles at the climax of Devonian greenhouse conditions may have contributed to the dynamics of flow of pulsed mineralizing fluids for sediment-hosted Cu-Co sulfide mineralization in reef-bearing carbonate platform.

## Supplementary Information

Below is the link to the electronic supplementary material.

Supplementary file1 (PDF 521 KB)
