# Supplementary material for: Metal-rich organic matter and hot continental passive margin: drivers for Devonian copper-cobalt-germanium mineralization in dolomitized reef-bearing carbonate platform
Source: Miner Depos. 2022 Jun 1;58(1):37–49. doi: 10.1007/s00126-022-01123-1 (PMC9829613; doi:10.1007/s00126-022-01123-1)
Supplement: Supplementary file 1 — Supplementary file1 (PDF 521 KB) [file 126_2022_1123_MOESM1_ESM.pdf]

Metal-rich organic matter and hot continental passive margin: Drivers for giant Devonian copper-cobalt-germanium mineralization in dolomitized reef-bearing carbonate platform

Nicolas J. Saintilan[1], Corey Archer[1], Colin Maden[1], Elias Samankassou[2], Stefano M. Bernasconi[3], David Szumigala[4], Zach Mahaffey[4], Andy West[4], Jorge E. Spangenberg[5]

[1] *Institute of Geochemistry and Petrology, ETH Zürich, Clausiusstrasse 25, 8092 Zürich, Switzerland*

[2] *Department of Earth Sciences, Rue des Maraîchers 13, 1205 Geneva, Switzerland*

[3] *Geological Institute, ETH Zürich, Sonneggstrasse 5, 8092 Zürich, Switzerland*

[4] *Ambler Metals LLC, 3700 Centerpoint Drive, Ste. #101, Anchorage, Alaska, USA*

[5] *Institute of Earth Surface Dynamics, University of Lausanne, Building Geopolis, 1015 Lausanne, Switzerland*

METHODS

Petrography and preparation of monophasic sulfide mineral separates. A total of six pyrite-, carrollite- and/or bornite-mineralized samples from the Cu-Co Bornite deposit, Alaska, USA, were selected (Table 1). The paragenetic sequence of the various sulfides was established based on macroscopic descriptions and microscopic petrographical observations of polished thin sections using transmitted and reflected light microscopy. The paragenetic relationships permitted to constrain the workflow for optimum mineral separation of individual sulfides into monophasic mineral separates according to the protocol presented in Saintilan et al. (2020). This workflow using 70–200 mesh size fractions combines the stepwise use of a Frantz Isodynamic Separator (FIS) and, additional treatment by heavy liquid separation of the magnetic (M) and non-magnetic (NM) fractions obtained at a given

current. For samples ASK-01, -03, -04, -05, bornite was isolated in the M0.72 fractions (i.e., fraction of magnetic material at an applied current of 0.72 amp) before purification from remaining gangue mineral by heavy liquid separation. For sample ASK-02, pyrite was concentrated in the NM1.5 fraction from the heavy fraction obtained by heavy liquid separation from the bulk 70–200 mesh size fraction. For sample ASK-06, bornite was isolated in the M0.72 fraction before purification from remaining gangue mineral by heavy liquid separation. Carrollite and trace chalcocite were collected in the NM1.7 fraction in this sample. The side slope of the FIS was adjusted to 10º and the NM1.7 fraction was further treated at 1.7 amp in order to remove trace chalcocite into the $M_{10}1.7$ fraction and to obtain a pure carrollite mineral separate in the $NM_{10}1.7$ fraction. Quality control of the final mineral separates was conducted according to the protocol by Saintilan et al. (2020).

Rhenium-osmium radiogenic isotope geochemistry. For each analysis, between 53 and 81 mg of pyrite, carrollite, or bornite mineral separates were weighed and transferred into thick-walled borosilicate Carius tubes (Shirey and Walker 1995). Each sulfide aliquot was dissolved in inverse Aqua Regia (~3 mL of 11N HCl and ~ 6 mL 16N HNO_3) with a known amount of "^{185}Re+^{190}Os spike" solution at 210ºC for 24h (Laboratory of Rhenium-Osmium Isotope Geochemistry and Geochronology, Isotope Geochemistry and Cosmochemistry Group, Institute of Geochemistry and Petrology, ETH Zürich). The laboratory protocol used in the present work is described in full details in Selby and Creaser (2001), Selby et al. (2009), Hnatyshin et al. (2016), and Li et al. (2017). In brief, Os was isolated and purified from the inverse Aqua Regia solution by chloroform ($CHCl_3$)-hydrobromic acid (HBr) solvent extraction at room temperature, and, by microdistillation (Roy-Barman and Allègre 1995; Cohen et al. 1996; Shen et al. 1996; Birck et al. 1997; Selby and Creaser 2001). The Re was isolated using an acetone-sodium hydroxide (Acetone-NaOH) step (Matthews and Riley

1970; Bozkhov et al. 1985; Li et al. 2009), followed by HCl–HNO_3-based anion chromatography (Morgan et al. 1991; Cumming et al. 2013). The Re and Os isotopic compositions were determined by negative thermal ionization mass spectrometry (N-TIMS) using a Thermo Scientific Triton mass spectrometer at the Institute of Geochemistry and Petrology, ETH Zürich. Rhenium and Os were loaded onto outgassed Ni and Pt filaments, respectively. Rhenium was measured as ReO_4^- in static mode on Faraday collectors, whereas Os was measured as OsO_3^- in peak-hopping mode on a single electron multiplier (Creaser et al. 1991; Völkening et al. 1991). Measurement quality was monitored by repeated measurements of in-house Re (125 pg aliquot – $^{185}Re/^{187}Re = 0.59860 \pm 0.00051$, 2σ, $n = 22$) and Os (Durham Romil Os Standard, DROsS; Nowell et al. 2008; 50 pg aliquot – $^{187}Os/^{188}Os = 0.16092 \pm 0.00069$, 2σ, $n = 12$) standard solutions. Total procedural blank was 1.82 ± 0.32 pg Re, 34 ± 20 fg Os with a blank $^{187}Os/^{188}Os$ isotope composition using inverse Aqua Regia of 1.77 ± 1.60 (2σ, $n = 3$) for batches RO-004 to RO-006. For batches RO-007 and RO-008, total procedural blank was 2.30 ± 0.18 pg Re, 44 ± 2 fg Os with a blank $^{187}Os/^{188}Os$ isotope composition using inverse Aqua Regia of 0.43 ± 0.08 (2σ, $n = 2$). The analytical uncertainties result from full error propagation of weighing errors, spike calibration, standard measurements, mass spectrometry analyses and blanks.

Calculation and validation of isochron and model Re-Os ages. *Common* Os corresponds to any isotope of Os, such as the stable normalizing isotope ^{188}Os and the most naturally abundant isotope ^{192}Os, that is incorporated by sulfide species at the time of mineralization in the environment of precipitation and prior to closure of the Re-Os isotopic system and initiation of atomic decay of ^{187}Re (Stein et al. 2000; Morelli et al 2005). In all analyzed sulfides, ^{192}Os, which is a robust approximation of the total *common* Os content, represents only a very small fraction of total Os (11 to 113 pg g^{-1} ^{192}Os). As a result, all aliquots of

bornite, carrollite and pyrite have elevated $^{187}Re/^{188}Os$ values (9,615–13,521) and highly radiogenic $^{187}Os/^{188}Os$ ratios (59.3–87.5). Such $^{187}Re/^{188}Os$ values and $^{187}Os/^{188}Os$ ratios are diagnostic of a total ^{187}Os budget, after blank correction, that largely comprises radiogenic ^{187}Os ($^{187}Os^{*}$; 95.0–98.8%) with negligible correction for ^{187}Os contributed by *common* Os (Table 2). This correction relies on the primary knowledge of the $^{187}Os/^{188}Os_{initial}$ value (called Os_i) for *common* Os. The $^{187}Os/^{188}Os$ values of individual aliquots are isotopically explained as follows in Equation 1 where λ is the decay constant of ^{187}Re as determined by Smoliar et al. (1996; $\lambda = 1.666e^{-11} \pm 5.165e^{-14}$ a^{-1}, 2σ), and t is the age of mineralization:

$$(^{187}Os/^{188}Os)_{measured} = (^{187}Os/^{188}Os)_{initial} + (^{187}Re/^{188}Os)_{measured} \cdot (e^{\lambda * t} - 1) \qquad (1);$$

$$(^{187}Os/^{188}Os)_{measured} - (^{187}Os/^{188}Os)_{initial} = (^{187}Re/^{188}Os)_{measured} \cdot (e^{\lambda * t} - 1) \qquad (1');$$

To account for the Os_i and to exploit its potential in understanding the greater geodynamic environment at the time of sulfide mineralization, Re-Os data for bornite are plotted in the $^{187}Os/^{188}Os$ vs. $^{187}Re/^{188}Os$ space with the error correlation value (*rho;* Ludwig 1980) and the 2σ calculated uncertainties for the $^{187}Os/^{188}Os$ and $^{187}Re/^{188}Os$ values. An isochron date is calculated using IsoplotR with the *Model 3* regression rationale to account for potential variability in the initial Os_i of each aliquot (Vermeesch 2018). With the estimate of Os_i and its uncertainty, individual model ages t for each bornite aliquot are calculated using Equation (2) derived from (1'):

$$^{187}Os^{*} = {}^{187}Re \cdot (e^{\lambda * t} - 1) \rightarrow t = (1/\lambda) \cdot \ln(^{187}Os^{*}/^{187}Re + 1) \quad (2);$$

An estimate of the $^{187}Os/^{188}Os$ isotope composition of the primitive upper mantle at 378 Ma is calculated by using present-day values of $^{187}Re/^{188}Os = 0.435 \pm 0.055$ and $^{187}Os/^{188}Os = 0.130 \pm 0.001$ for the primitive upper mantle (Meisel et al. 2001; Carlson 2005).

Given the limited number of data for carrollite for a single sample in which carrollite was available, we utilize the range of Os_i values from the isochron regression of the six bornite aliquots as a representative estimate of the $^{187}Os/^{188}Os$ isotope composition of *common* Os in the entire system at the time of Cu-Co mineralization. We can then correct for negligible initial *common* ^{187}Os abundances in carrollite (>98% $^{187}Os^*$) and determine the abundances in radiogenic $^{187}Os^*$ for all four aliquots. We follow the same rationale for the three aliquots of fine-grained pyrite cement (>95% $^{187}Os^*$) from a single sample (ASK-02). Thus, we can also calculate preliminary individual model ages *t* for each carrollite and pyrite aliquot using Equation (2).

Sulfur stable isotope geochemistry. Sulfur isotope geochemistry was carried out at the Geological Institute at ETH Zürich (samples ASK-01, -03, -04, -06) and at the Institute of Earth Surface Dynamics, University of Lausanne (samples ASK-02, -05, -06), Switzerland. About 5 to 10 mg of pure bornite, carrollite, pyrite mineral separates were powdered in a hand-held agate mortar and pestle. Sulfide aliquots were analyzed by standard techniques using protocols by Bernasconi et al. (2017) at ETH Zürich and by Robinson and Kusakabe (1975) at the University of Lausanne. Analyses of standards distributed by the International Atomic Energy Agency (IAEA-S_1, -S_2, and -S_3) were performed for calibration. Repeated measurement of these standards gave reproducibility better than 0.2‰ at ETH Zürich and 0.3‰ at the University of Lausanne. Data are reported in $\delta^{34}S$ notation as per mil (‰) variations from the Vienna Cañon Diablo Troilite (V-CDT) standard.

Molybdenum stable isotope geochemistry. All procedures of molybdenum isotope geochemistry were carried out at the Institute of Geochemistry and Petrology at ETH Zürich. Glass scintillation vials (22mL) with PTFE-lined lids were refluxed individually with a few milliliters of 1M HCl for 48h, prior to undergoing 3 rinses with ultrapure Milli-Q® water (18.2 MΩ) and a final reflux in Milli-Q® water for 24h. Each vial was rinsed and dried. Between 21 and 77 mg of monophasic sulfide mineral separate (bornite/carrollite/pyrite) was added to each vial and dissolved in a mixture of 1 mL 11N HCl and 3 mL 16N HNO_3 at 95ºC. After 24h, vials were uncapped and solutions taken to dryness. Each dried cake was completely dissolved by adding 1 mL of double-distilled 1N HCl to each vial. Aliquots (20 µL) from each vial were taken to dryness and diluted in 2% HNO_3. Molybdenum (Mo) and nickel (Ni) concentrations were measured on a Thermo Fisher Scientific Element XR sector-field inductively coupled plasma mass spectrometer (ICP-MS). The total blanks of those successive procedures totaled 1.5 to 2.5 ng g^{-1}. Given the total Mo concentrations of the sulfide samples between 3 and 62 µg g^{-1}, those blank values were considered negligible and not corrected for here.

For each sample, based on the previously determined Mo contents, aliquots of between 22 and 470 µL of solution approximating 100 ng of Mo were mixed with an appropriate amount of ^{100}Mo–^{97}Mo double-spike (Archer and Vance 2008) prior to chemical purification. Mo was purified using previously described protocols (Archer and Vance 2008; Bura-Nakić et al. 2018; He et al. 2021). The purified Mo cuts were taken to dryness and then re-dissolved in 2% HNO_3 for mass spectrometry. The Mo fractions were analyzed for isotope composition using a Thermo Fisher Scientific Neptune Plus multi collector ICP-MS in low-resolution mode. Samples were introduced in 2% (v/v; ~ 0.3 M) HNO_3 solution via Savillex C-Flow PFA nebulizer (50 µL min^{-1}) attached to a Teledyne-Ceta Aridus II desolvator. Instrumental mass fractionation was corrected for using the double spike method as previously described (Siebert

et al. 2001; Archer and Vance 2008). Measurements were performed using eight Faraday collectors in static mode on masses 91, 92, 94, 95, 96, 97, 98, 99, and 100 simultaneously, though only masses 95, 97, 99 and 100 are used in the double spike data reduction algorithm. The presence of ruthenium (Ru) is monitored and corrected for within run using mass 99. Again, although not utilized, the presence of zirconium (Zr) interferences is monitored by using mass 91 (Siebert et al. 2001; Archer and Vance 2008). Molybdenum isotope ratios are presented in the standard delta notation, where $\delta^{98/95}Mo$ (‰) = $[^{98}Mo/^{95}Mo_{sample}/^{98}Mo/^{95}Mo_{standard} - 1] \times 1000$. All Mo isotope compositions for samples are reported relative to NIST SRM 3134 = +0.25‰ (Nägler et al. 2014). The Mo double spike method was verified and long-term reproducibility assessed via the analysis of an in-house CPI standard. Over a five-year period, including the period of this study, analysis of our in-house CPI standard with standard/spike ratios in the range of 0.5 to 2 gave $\delta^{98/95}Mo = 0.02 \pm 0.05$‰ (all isotope data reported as 2 standard deviations) relative to NIST SRM 3134 = +0.25‰ (Siebert et al. 2003; Nakagawa et al. 2012). The procedural blank added during Mo purification was conservatively determined to be ≤ 100 pg, and is therefore negligible to any blank correction.

REFERENCES

Archer C and Vance D (2008) The isotopic signature of the global riverine molybdenum flux and anoxia in the ancient oceans. Nat. Geoscience, 1:597-600.

Bernasconi SM, Meier I, Wohlwend S, Brack P, Hochuli PA, Bläsi H, Wortmann UG, Ramseyer K (2017) An evaporite-based high-resolution sulfur isotope record of Late Permian and Triassic seawater sulfate. Geochim. Cosmochim. Acta, 204:331-349.

Birck JL, Roy-Barman M, Capmas F (1997) Re-Os isotopic measurements at the femtomole level in natural samples. Geostandard Newsletter, 21:19-27.

Bozkhov OD, Jordanov N, Borissova LV, Fabelinskii YI (1985) Extraction-spectral emission determination of traces of rhenium using ICP. Fres. Zeitschr. Anal. Chem., 321:453-456.

Bura-Nakić E, Andersen MB, Archer C, de Souza GF, Marguš M, Vance D (2018) Coupled Mo-U abundances and isotopes in a small marine euxinic basin: Constraints on processes in euxinic basins. Geochim. Cosmochim. Acta, 222:212-229.

Carlson RW (2005) Application of the Pt-Re-Os isotopic systems to mantle geochemistry and geochronology. Lithos, 82:249-272.

Cohen AS, Waters FG (1996) Separation of osmium from geological materials by solvent extraction for analysis by thermal ionisation mass spectrometry. Anal. Chim. Acta, 332:269-275.

Creaser RA, Papanastassiou DA, Wasserburg GJ (1991) Negative thermal ion mass spectrometry of osmium, rhenium and iridium. Geochim. Cosmochim. Acta, 55:397–401.

Cumming VM, Poulton SW, Rooney AD, Selby D (2013) Anoxia in the terrestrial environment during the late Mesoproterozoic. Geology, 41:583-586.

He Z, Clarkson MO, Andersen MB, Archer C, Huang F, Vance D (2021) Temporally and spatially dynamic redox conditions on an upwelling margin: the impact on coupled sedimentary Mo and U isotope systematics, and implications for the Mo-U paleoredox proxy. Geochim. Cosmochim. Acta, 309:251-271.

Hnatsyhin D, Kontak DJ, Turner EC, Creaser RA, Morden R, Stern RA (2016) Geochronologic (Re-Os) and fluid-chemical constraints on the formation of the Mesoproterozoic-hosted Nanisivik Zn-Pb deposit, Nunavut, Canada: Evidence for early diagenetic, low-temperature conditions of formation. Ore Geol. Rev., 79:189-217.

Li C, Qu W, Du A, Sun W (2009) Comprehensive study on extraction of rhenium with acetone in Re-Os isotopic dating. Rock Miner. Anal., 28:233-238 (in Chinese, with English abstract).

Li Y, Selby D, Condon D, Tapster S (2017) Cyclic magmatic-hydrothermal evolution in porphyry systems: High-precision U-Pb and Re-Os geochronology constraints on the Tibet Qulong porphyry Cu-Mo deposit. Econ. Geol., 112:1419-1440.

Ludwig KR (1980) Calculation of uncertainties of U-Pb isotope data. Earth Planet. Sci. Lett., 46:212-220.

Matthews A, Riley J (1970) The determination of rhenium in seawater. Anal. Chim. Acta, 51:483-488.

Meisel T, Walker RJ, Irving AJ, Lorand JP (2001) Osmium isotopic compositions of mantle xenoliths: a global perspective. Geochim. Cosmochim. Acta, 65:1311-1323.

Morelli RM, Creaser RA, Selby D, Kontak DJ, Horne RJ (2005) Rhenium-osmium geochronology of arsenopyrite in Meguma Group gold deposits, Meguma Terrane, Nova Scotia, Canada: Evidence for multiple gold-mineralizing events. Econ. Geol., 100:1229-1242.

Morgan JW, Golightly DW, Dorrzapf AF (1991) Methods for the separation of rhenium, osmium and molybdenum applicable to isotope geochemistry. Talanta, 38:259-265.

Nägler TF, Anbar AD, Archer C, Goldberg T, Gordon GW, Greber ND, Siebert C, Sohrin Y, Vance D (2014) Proposal for an international molybdenum isotope measurement standard and data interpretation. Geostand. Geoanal. Research, 38:149-151.

Nakagawa Y, Takano S, Firdaus ML, Norisuye K, Hirata T, Vance D, Sohrin Y (2012) The molybdenum isotopic composition of the modern ocean. Geochem. J. , 46:131.

Nowell GM, Pearson DG, Parman SW, Luguet A, Hanski E (2008) Precise and accurate $^{186}Os/^{188}Os$ and $^{187}Os/^{188}Os$ measurements by multi-collector plasma ionisation mass spectrometry, part II: Laser ablation and its application to single-grain Pt-Os and Re-Os geochronology. Chem. Geol., 248:394-426.

Robinson BW, Kusakabe M (1975) Quantitative preparation of sulfur dioxide for $^{34}S/^{32}S$ analyses from sulfides by combustion with cuprous oxide. Anal. Chem., 47:1179-1181.

Roy-Barman M, Allègre CJ (1995) $^{187}Os/^{186}Os$ in oceanic island basalts: tracing oceanic crust recycling in the mantle. Earth Planet. Sci. Lett., 129:145-161.

Saintilan NJ, Selby D, Hughes JW, Schlatter D, Kolb J, Boyce A (2020) Mineral separation protocol for accurate and precise rhenium-osmium (Re-Os) geochronology and sulfur isotope composition of individual sulfide species. MethodsX, 7:100944.

Selby D, Creaser RA (2001) Re-Os geochronology and systematics in molybdenite from the Endako porphyry molybdenum deposit, British Columbia, Canada. Econ. Geol., 96:197-204.

Selby D, Kelley KD, Hitzman MW, Zieg J (2009) Re-Os sulfide (bornite, chalcopyrite, and pyrite) systematics of the carbonate-hosted copper deposits at Ruby Creek, southern Brooks Range, Alaska. Econ. Geol., 104:437–444.

Shen JJ, Papanastassiou DA, Wasserburg GJ (1996) Precise Re-Os determinations and systematics of iron meteorites. Geochim. Cosmochim. Acta, 60:2887-2900.

Shirey SB, Walker RJ (1995) Carius tube digestion for low-blank rhenium-osmium analysis. Anal. Chem., 67:2136–2141.

Siebert C, Nägler TF, Kramers JD (2001) Determination of molybdenum isotope fractionation by double-spike multicollector inductively coupled plasma mass spectrometry. Geochem. Geophys. Geosyst., 2:2000GC000124.

Siebert C, Nägler TF, von Blanckenburg F, Kramers JD (2003) Molybdenum isotope records as a potential new proxy for paleoceanography. Earth Planet. Sci. Lett., 211:159-171.

Smoliar MI, Walker RJ, Morgan JW (1996) Re-Os ages of group IIA, IIIA, IVA, and IVB iron meteorites. Science, 271:1099–1102.

Stein HJ, Morgan JW, Scherstén A (2000) Re-Os dating of low-level highly radiogenic (LLHR) sulfides: The Harnäs gold deposit, Southwest Sweden, records continental-scale tectonic events. Econ. Geol., 95:1657-1671.

Vermeesch P (2018) IsoplotR: a free and open toolbox for geochronology. Geosci. Frontiers, 9:1479-1493.

Völkening J, Walczyk T, Heumann K (1991) Osmium isotopic ratio determination by negative thermal ionization mass spectrometry. Inter. J Spectro. Ionic Phys., 105:147–159.

Supplementary Figure SI 1. Rhenium-osmium geochronology results for bornite, carrollite and pyrite in the studied samples a. Model 3 Re-Os isochron regression for bornite in the $^{187}Os/^{188}Os$ vs. $^{187}Re/^{188}Os$ space. Individual ellipses show 2σ uncertainty of each data point in $^{187}Os/^{188}Os$ vs. $^{187}Re/^{188}Os$ space. Ellipses are constructed from maximum and minimum error vectors that are orthogonal to each other. Maximum and minimum uncertainties are statistical values that are calculated from the uncertainties of the $^{187}Os/^{188}Os$ and $^{187}Re/^{188}Os$ ratios for a given data point. Final uncertainties were calculated by full error propagation of uncertainties in the Re and Os measurements, blank values, isotopic compositions, spike calibrations, and reproducibility of the standard Re and Os values. The error correlation function, *rho*, is utilized for isochron regression (Ludwig 1980). Isochron uncertainty includes uncertainty in the ^{187}Re decay constant (Smoliar et al. 1996). Excess scatter beyond the reported analytical uncertainties is indicated by a mean square weighted deviation (MSWD) at 5.3. The limited spread in $^{187}Re/^{188}Os$ values and variability in the Os_i (2.40 ± 2.86) during bornite precipitation may explain this scatter (see text for details). The inset shows the individual Os_i calculated for each aliquot at an age of 378 ± 15 Ma. Estimates of the $^{187}Os/^{188}Os$ isotopic composition of the primitive upper mantle at 378 Ma (calculated using $^{187}Re/^{188}Os$, $^{187}Os/^{188}Os$ values for the present-day primitive upper mantle in Meisel et

al. 2001 and Carlson 2005) and the $^{187}Os/^{188}Os$ of Upper Devonian seawater in open-marine setting (Saintilan et al. 2021) are shown for reference. **b**. Preliminary Re-Os model ages for individual aliquots of carrollite and their weighted mean average. **c**. Preliminary Re-Os model ages for individual aliquots of pyrite and their weighted mean average.

ESM Table 1. Samples ID with mineral of interest for analysis in the present study (Coordinates according to Universal Transverse Mercator, Zone 4 coordinate system NAD83 Datum).

Samples	Borehole ID	Easting	Northing	Depth (m)	Mineral(s) analysed in this study
ASK-01	RC-18-0252	589'867.80	7'440'015.37	439.50	Bornite - Cu_5FeS_4
ASK-02	RC-11-0186	589'113.25	7'439'746.00	25.27	Pyrite - FeS_2
ASK-03	RC-11-0187	590'273.50	7'440'008.00	491.06	Bornite - Cu_5FeS_4
ASK-04	RC-18-0247	590'536.75	7'440'675.80	796.25	Bornite - Cu_5FeS_4
ASK-05	RC-18-0254	589'883.86	7'440'798.93	690.75	Bornite - Cu_5FeS_4
ASK-06	RC-18-0187	590'273.50	7'440'008.00	489.57	Bornite - Cu_5FeS_4 & Carrollite - $CuCo_2S_4$

ESM Table 2. Repeats of molybdenum isotope data for bornite, carrollite and pyrite at Ruby Creek-Bornite, Southern Brooks Range, Alaska.

Sample	Mineral	Petrographic texture	Sample weight	$\delta^{98/95}$Mo V-NIST$_{SRM3134}$ (Batch #1)	$\delta^{98/95}$Mo V-NIST$_{SRM3134}$ (Repeat batch #1)	$\delta^{98/95}$Mo V-NIST$_{SRM3134}$ (Batch #2)
			(g)	(‰)	(‰)	(‰)
ASK-02a	Pyrite - FeS_2	cement of ferroan dolomite	0.03714	+ 2.22	+ 2.24	+ 2.23
ASK-02b	Pyrite - FeS_2		0.07538	+ 2.13	+ 2.09	+ 2.04
ASK-06_CAR_a	Carrollite - $Cu[Co,Ni]_2S_4$	cementing solid bitumen and fragments of dolomitised carbonaceous limestone	0.07432	+ 3.78	+ 3.86	+ 3.87
ASK-06_CAR_b	Carrollite - $Cu[Co,Ni]_2S_4$		0.05320	+ 3.86	+ 3.88	+ 3.89
ASK-01	Bornite - Cu_5FeS_4	replacing coarse-grained pyrite and associated with solid bitumen	0.02899	+ 2.44	+ 2.44	+ 2.48
ASK-03	Bornite - Cu_5FeS_4	cementing solid bitumen and fragments of dolomitised carbonaceous limestone	0.02114	+ 5.48	+ 5.49	+ 5.48
ASK-04	Bornite - Cu_5FeS_4	associated with white hydrothermal dolomite and cementing clasts of dolomite matrix	0.02856	+ 2.75	+ 2.70	+ 2.72
ASK-05	Bornite - Cu_5FeS_4	associated with white hydrothermal dolomite	0.06387	+ 2.21	+ 2.21	+ 2.21
ASK-06_Bn	Bornite - Cu_5FeS_4	cementing solid bitumen and fragments of carbonaceous limestone	0.03934	+ 3.53	+ 3.58	+ 3.58

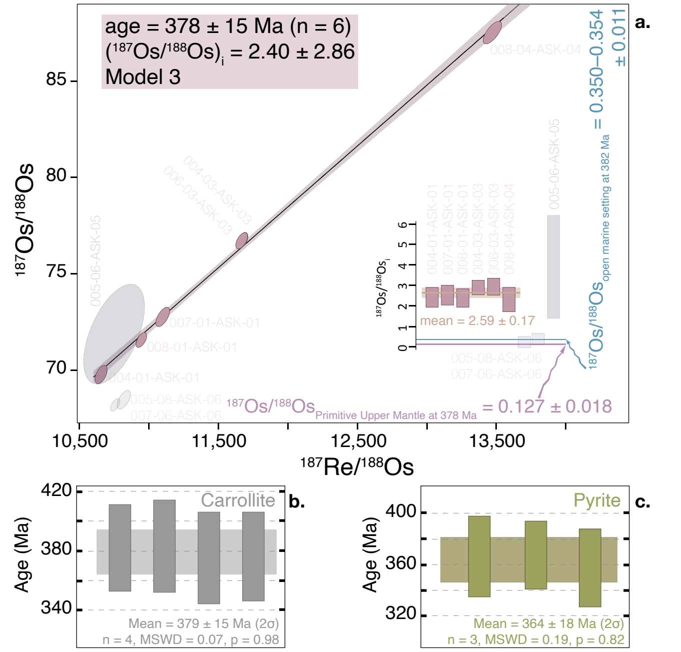

age = 378 ± 15 Ma (n = 6)
($^{187}Os/^{188}Os)_i$ = 2.40 ± 2.86
Model 3
a.
$^{187}Os/^{188}Os$
$^{187}Re/^{188}Os$
85
80
75
70
10,500
11,500
12,500
13,500
008-04-ASK-04
004-03-ASK-03
006-03-ASK-03
005-06-ASK-05
007-01-ASK-01
008-01-ASK-01
004-01-ASK-01
005-08-ASK-06
007-06-ASK-06
$^{187}Os/^{188}Os_i$
004-01-ASK-01
007-01-ASK-01
008-01-ASK-01
004-03-ASK-03
006-03-ASK-03
008-04-ASK-04
mean = 2.59 ± 0.17
$^{187}Os/^{188}Os_{open\ marine\ setting\ at\ 382\ Ma}$ = 0.350–0.354 ± 0.011
$^{187}Os/^{188}Os_{Primitive\ Upper\ Mantle\ at\ 378\ Ma}$ = 0.127 ± 0.018
b.
Age (Ma)
420
380
340
Carrollite
Mean = 379 ± 15 Ma (2σ)
n = 4, MSWD = 0.07, p = 0.98
c.
Age (Ma)
400
360
320
Pyrite
Mean = 364 ± 18 Ma (2σ)
n = 3, MSWD = 0.19, p = 0.82
